# Assessment of knowledge, attitude and practice on tuberculosis among teacher trainees of Samtse College of Education, Bhutan

**DOI:** 10.1371/journal.pone.0241923

**Published:** 2020-11-06

**Authors:** Thinley Dorji, Tandin Tshering, Kinley Wangdi

**Affiliations:** 1 Kanglung Hospital, Trashigang, Bhutan; 2 Samtse College of Education, Samtse, Bhutan; 3 Department of Global Health, Research School of Population Health, College of Health and Medicine, Australian National University, Acton, Canberra, ACT, Australia; Imam Abdulrahman Bin Faisal University, SAUDI ARABIA

## Abstract

**Background:**

Tuberculosis (TB) is a major public health problem in Bhutan despite the implementation of directly observed treatment short-course since 1997. This study aimed to assess the knowledge, attitude and practice on TB among the teacher trainees of Samtse College of Education.

**Methodology:**

A cross-sectional study was conducted among the teacher trainees of Samtse College of Education. A standardized pretested questionnaire was distributed and self-administered. The participants were selected randomly using randomization. The data was entered in Epi-data 4.4.2.1 and analysed using STATA version 13. A score of 1 point for a correct answer and 0 for wrong/don’t know answer was given. The knowledge, attitude and practice score were divided into good and poor based on 50% cut off. Logistic regression was used for the analysis to identify the significant covariates.

**Results:**

A total of 420 trainees responded to the survey questionnaire. The average knowledge score on TB was 10.7 (Range = 0–21). Two hundred and forty respondents (58.6%) had low knowledge (mean score 7.8±2.5) on TB. Cough, chest pain and weight loss were correctly reported as the symptoms of TB by 306 (72.9%), 187(44.5%) and 187 (44.5%) participants. Eighty-nine-point five percent (376) of participants reported coughing as the main route of TB transmission and 85% (357) of the participants said that it could be prevented by covering the mouth while coughing. In multivariable analysis; the trainees in the junior years of college had good knowledge of TB compared with the senior years (adjusted odds ratio [AOR] 2.02; 95% confidence interval [CI] 1.18–3.5; p-value 0.011). Respondents previously treated for TB were more likely to have good knowledge on TB compared to those who never had TB in the past (AOR 2.39; 95% CI 1.07–5.31; p-value 0.033). The majority (93%) of respondents had a good attitude towards TB cases. Female trainees were 2.4 (95% CI 1.02–5.62; p-value 0.045) times more likely than male trainees to have a positive attitude towards TB. Eighty-eight percent of the respondents reported that they would visit the hospital if they had TB symptoms. The mean score for the practice on TB was 1.33±0.59 (Range:0–2).

**Conclusion:**

In this study, the majority of the trainees had poor knowledge on TB, especially among the trainees in senior years of college and those who had never suffered from TB. The attitude towards TB was good especially among the female trainees. However, the overall practice was poor among the participants. Therefore, the Ministry of Health should collaborate with relevant stakeholders especially the Ministry of Education to incorporate topics on TB in the syllabus of students and colleges to create awareness on it.

## Introduction

The United Nation’s Sustainable Development Goals (SDG) “Health goal number 3” plans to end tuberculosis (TB) epidemic by 2030 [1]. However, this ambitious plan could be at risk as TB still infects millions globally every year especially in the developing countries of Asia and Africa [2]. According to the World Health Organization (WHO), TB infected 10.4 million people and caused an estimated 1.45 million deaths (including both in HIV negative and positive patients) in 2018 [2]. Although the number of people dying from TB has started to decrease since the introduction of directly observed treatment short-course (DOTS), it still is one of the top causes of mortality [3].

In Bhutan, TB continues to be one of the leading public health diseases with an incidence rate of 149 per 100,000 population [2] despite the introduction of DOTS in 1997 [4]. There have been significant efforts to combat TB by the Ministry of Health through programs such as training of health workers on TB management, creating public awareness during the World TB Day and education using mass media. Despite these efforts, past studies in other countries identified delayed treatment-seeking for TB as one of the main reasons for the spread of TB in the communities [5, 6]. Expanding testing, improved surveillance, screening and treatment of TB is critical in achieving the global goal of TB elimination by 2030 [7].

A risk estimation study in the Bhutanese population reported that the annual risk of TB infection is low [8]. Despite this, the current challenge of the National TB Control Program (NTCP) is to improve and bridge the case detection gaps for early diagnosis and prompt initiation of treatment [11]. One of the reasons for delayed diagnosis was visit to local healers before seeking help from health centres [9]. In 2019, a total of 946 TB cases diagnosed in Bhutan [10]. Of this, more than 85% of the cases were reported by eight districts [11].

Colleges and schools can be a potential source of disease transmission including TB due to the crowded environment and high level of person-to-person contact [12, 13]. Earlier studies have reported outbreaks of TB among students in Italy and Ethiopia [14, 15] due to repeated exposure with the TB cases not under treatment. Past studies have shown that poor knowledge on TB leads to a delay in seeking care for TB [16, 17]. A person with an active TB infects on an average of 10–15 people per year [18]. This highlights the need for early case detection and treatment of all TB cases.

In Bhutan, the education is provided free of cost by the Royal Government of Bhutan. Although science subjects are taught in schools, the curriculum does not include teachings on infectious diseases including TB. A nationwide study in Bhutan showed that 14% of the total TB patients in Bhutan were under the age of 15 years [19]. This is an alarming figure that needs attention. One of the reasons could be lack of knowledge and understanding on TB among the students. Therefore, understanding of knowledge, attitude and practice (KAP) on TB is crucial especially among those that play a key role in the society. The findings from this study could be used to improve the KAP on TB amongst the future teachers because they can use this knowledge for early referral of students from their schools. Moreover, educating students on TB can help in the dissemination of the knowledge on TB in the community. This can improve the health seeking behaviour for TB, thereby halting the ongoing transmission. Therefore, the study aimed to understand the KAP on TB among the teacher trainees of Samtse College of Education, Bhutan.

## Materials and methods

### Study area

The study was conducted in Samtse College of Education from September 1^st^ to September 30^th^, 2019. The college is located in the Southern part of Bhutan where the burden of TB is high [11]. It is one of the only two teacher training institutes in Bhutan. The college offers a bachelor of education for primary and secondary education along with masters of education for science, geography and English. There were a total of 916 trainees with 52% of them males in 2019.

### Study design and sample size

A cross-sectional study was conducted among the trainees of Samtse College of Education. The sample size was calculated using the formula, n = $\frac{z^{2}p(100-p)}{d^{2}}$ [20] (where n = sample size, z = confidence level for normal distribution, p = estimated proportion and d = absolute error). Taking a confidence interval (CI) of 95% interval, with a probability of 50% and margin of error at 5% and a non-response rate of 10%, the sample size was rounded off to 425. The participants for this study were selected randomly using computer-generated randomization from the student database.

### Inclusion criteria

The inclusion criteria for this study included: (I) currently enrolled teacher trainees and (II) trainees willing to sign an informed consent form.

### Data collection tools

The data was collected using a standardized pretested questionnaire adapted from the WHO guidebook for conducting KAP studies on TB [21]. The questionnaire was initially pre-tested among 20 trainees and modified accordingly to the feedback. This group of trainees were latter excluded from the study. The questionnaire was randomly distributed among the trainees and the responses were self-administered.

The questionnaire was divided into two parts. The first part comprised of socio-demographic characteristics and the second part included questions on knowledge, attitudes and practices. The questionnaire consisted of both multiple-choice questions with a single as well as multiple answers.

The knowledge section had 23 correct answers. Each correct item was scored “1” and “0” for incorrect or don’t know responses. There were six questions for attitudes and two questions for practice. For both attitude and practice response, a score of “1” was given for favourable attitude and practice and “0” for other responses. The outcome was divided into two strata: good if the final score was above 50% of the total score and poor otherwise [22].

### Statistical analysis

The data were entered in Epi-data 4.4.3.1 (EpiData Association, Denmark) and analysed using STATA version 13 (Stata Corporation, College Station, TX, USA) software. Descriptive statistics (frequencies and percentages) were used to describe the demographic characteristics. Univariate and multivariate logistic regression was performed to check for associations with the independent variables. A p-value < 0.05 was considered significant.

### Ethical approval and confidentiality

The ethical approval was provided by the Research Ethics Board of Health (REBH), Ministry of Health, Bhutan (REBH/Approval/2019/016). In addition, an administrative clearance was obtained from the college administration. All the participants signed an informed consent form before participating in this study. The survey data are protected using a password encrypted folder in the principal investigator’s and co-investigators’ computers.

## Results

### Socio-demographic characteristics

**Fig 1:**
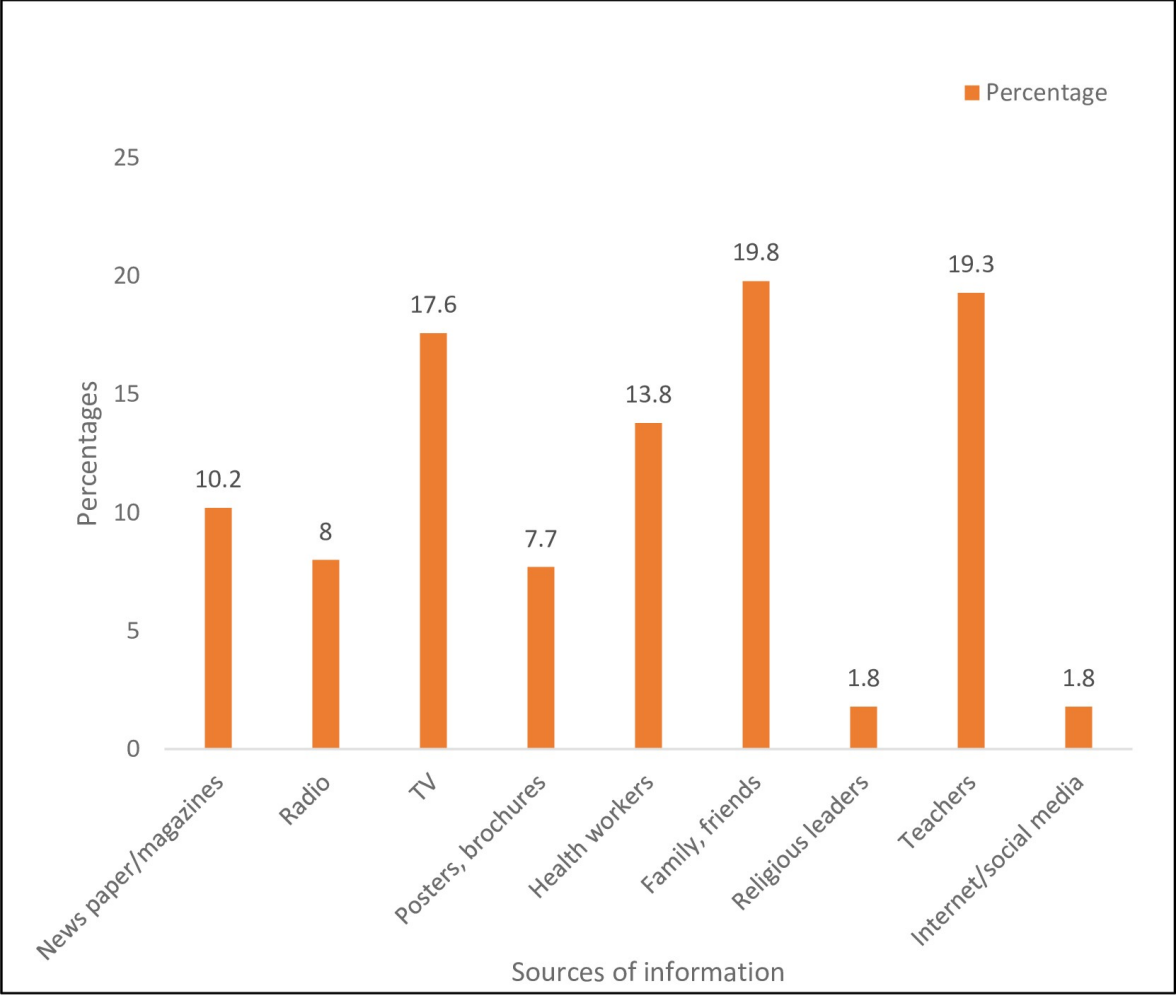
Sources of TB information.

Out of 425 participants invited for the survey, the response rate was 98.8% (n = 420). There was an almost equal number of male and female participants (46.9% vs 53.1%) (Table 1). The mean age of the respondents was 23 years (SD 3.2; Range 18–41 years). More than half (60.7%) of the respondents reported that they never smoked and 43% had never consumed alcohol. The most common source of information on TB were family and friends, followed by teachers (Fig 1).

**Table 1:** Socio-demographic characteristic of study participants (n = 420).

Variables		Frequency	Percentage
**Age (years)**			
	<23 years	236	56.2
	≥ 23 years	184	43.8
**Education**			
	First year	17	4.1
	Second year	93	22.1
	Third year	166	39.5
	Fourth year	62	14.8
	PGDE	61	14.5
	Master in Education	21	5.0
**Sex**			
	Male	197	46.9
	Female	223	53.1
**Marital status**			
	Single	380	90.5
	Married	37	8.8
	Divorced	3	0.7
**Smoking**			
	Never used	255	60.7
	Past user	95	22.6
	Current user	70	16.7
**Alcohol use**			
	Never used	182	43.3
	Past user	133	31.7
	Current user	105	25.0

PGDE: Postgraduate diploma in Education

### Knowledge of TB symptoms, transmission and diagnosis

The overall mean knowledge score on TB cause, symptoms, diagnosis and treatment was 10.66 (Range 0–21). Two hundred and forty-six (58.6%) of the participants had low TB knowledge (score < 11.5) (mean score 7.8±2.5) while 174 (41.4%) had good knowledge on TB.

The participant’s response to TB knowledge are shown in Table 2. The majority of the trainees (60%) knew that TB was caused by a bacterium. With regards to TB symptoms, cough more than two weeks was reported by 73% of participants followed by chest pain (44%), weight loss (44%) and blood in the sputum (37%) respectively. The most common cause of TB transmission mentioned was coughing (89%). There was a misconception that TB could be transmitted by sharing of the dishes (15% of the respondents). The participants reported the two most common methods of TB diagnosis were blood test (40.3%) and sputum examination (33.4%). Almost 60% of the respondents correctly answered that the TB vaccine was available. More than half of the participants (58%) knew the duration of TB treatment to be 6–8 months. However, a small proportion (6%) of participants thought the duration of TB treatment to be 1–2 weeks.

**Table 2:** Knowledge on TB cause, symptoms, transmission and treatment.

Variables	Response categories	Frequency	Percentage
Cause of TB			
	Bacteria	252	60
	Virus	120	28.6
	Fungus	2	0.5
	Don’t know	46	10.9
Symptoms of TB			
	Cough ≥ 2 weeks	306	72.9
	Fever	116	27.6
	Weight loss	187	44.5
	Blood in sputum	155	36.9
	Chest pain	187	44.5
	Loss of appetite	163	38.8
	Fatigue	80	19.1
	Others	19	4.5
	Don’t know	47	11.2
TB transmission			
	Through air droplets during cough	376	89.5
	Through handshakes	35	8.3
	Through sharing dishes	62	14.8
	Through touching items	27	6.4
	Don’t know	28	6.7
	Others	12	2.9
Diagnosis of Pulmonary TB			
	Blood test	186	44.3
	Sputum test	144	34.3
	Chest X ray	36	8.6
	Urine/stool test	29	6.9
	Don’t know	25	5.9
TB vaccine is available			
	Yes	250	59.5
	No	40	9.5
	Don’t know	130	40
Duration of TB treatment			
	1–2 weeks	26	6.2
	1–2 months	36	8.6
	6–8 months	244	58.1
	1–2 years	35	8.3
	Don’t know	79	18.8

### Knowledge of TB prevention and risk factors

The responses on TB prevention and risk factors are summarised in Table 3. Over 85% of the participants correctly reported that TB could be prevented by covering mouth during coughing while 20% of the participants responded that it could be prevented through the intake of good nutrition. More than 66% thought that smoking was a risk factor for TB followed by malnutrition (29.8%) and alcohol consumption (25.7%). Forty-six percent of the respondents reported that TB drugs should not be discontinued after symptoms improve and 49% mentioned that doing so could lead to the drug-resistant TB. Almost 80% of the respondents correctly mentioned that re-infection with TB could occur.

**Table 3:** Knowledge on prevention and risk factors on TB.

Variables	Responses	Frequency	Percentage
How can we prevent TB?			
	Covering mouth when coughing	357	85
	Avoid handshakes	26	6.2
	Avoid sharing dishes	66	15.7
	Washing hands after touching items	56	13.3
	Closing windows	9	2.1
	Through good nutrition	87	20.7
	Don’t know	24	5.7
What are the risk factors for TB?			
	Smoking	278	66.2
	Alcohol	108	25.7
	Malnutrition	125	29.8
	HIV	29	6.9
	DM	14	3.3
	Don’t know	75	17.9
Who can be infected with TB?			
	Anybody	375	89.3
	Only poor people	7	1.7
	Only homeless people	4	1
	Only alcoholic/drug users	13	3.1
	Only people with HIV	4	1
	Prisoners	1	0.2
	Don’t know	16	3.8
Can TB affect organs other than lungs?			
	Yes	229	54.5
	No	62	14.8
	Don’t know	129	30.7
Should patient discontinue drugs once they feel well?			
	Yes	137	32.7
	No	196	46.8
	Don’t know	86	20.5
What happens on discontinuation?			
	Patient gets better	57	13.6
	Develops DR-TB	206	49.0
	Don’t know	157	37.4
Can re-infection with TB occur?			
	Yes	334	79.5
	No	22	5.2
	Don’t know	64	15.2

HIV-Human Immunodeficiency virus, DM-Diabetes mellitus, DR-TB-Drug resistant TB

Table 4 describes the multivariate analysis of socio-demographic characteristics to the knowledge of TB. Trainees in the junior years of college had a significantly higher level of knowledge compared to the senior trainees (AOR 2.02; 95% CI 1.18–3.5; p-value 0.011). Moreover, a trainee previously treated for TB was 2.4 times more likely (95% CI 1.07–5.31; p-value 0.033) to have good knowledge compared to those who had not been treated for TB.

**Table 4:** Factors associated with TB knowledge among trainees in Samtse College.

Knowledge score				Univariate analysis		Multivariate analysis	
Variables		Poor, n (%)	Good, n (%)	COR (95% CI)	p-value	AOR (95% CI)	p-value
**Age (years)**							
	<23 years	136 (55.3)	100 (57.5)	Ref		Ref	
	≥ 23 years	110 (44.7)	74 (42.5)	0.9 (0.62–1.35)	0.66	1.19 (0.74–1.92)	0.476
**Sex**							
	Female	125 (50.8)	98 (56.3)	1.25 (0.84–1.84)	0.27	1.45 (0.95–2.21)	0.084
	Male	121 (49.2)	76 (43.7)	Ref			
**Education**							
	Junior years	152 (61.8)	124 (71.3)	1.53 (1.01–2.33)	0.04*	2.02 (1.18–3.5)	0.011*
	Senior years	94 (38.2)	50 (28.7)	Ref		Ref	
**Marital status**							
	Single	224 (91.1)	156 (89.7)	Ref		Ref	
	Married/divorced	22 (8.9)	18 (10.3)	1.17 (0.61–2.26)	0.63	2 (0.91–4.37)	0.082
**Previously treated for TB**							
	Yes	11 (4.5)	18 (10.3)	2.47 (1.13–5.36)	0.023*	2.39 (1.07–5.31)	0.033*
	No	235 (95.5)	156 (89.7)				
**Family treated for TB**							
	Yes	45 (18.3)	38 (21.8)	1.25 (0.77–2.02)	0.37	1.16 (0.7–1.9)	0.57
	No	201 (81.7)	136 (18.2)	Ref		Ref	

*p-value <0.05

COR- crude odds ratio; AOR- adjusted odds ratio; CI- confidence interval

Junior years -First year, second year and third year; Senior Years-Final year, Post graduate, Master of Education

### Attitudes of trainees towards TB

The attitude of the respondents to TB patients is summarised in Table 5. Ninety-eight percent and 91% of the participants reported TB to be a serious illness and a public health problem in Bhutan. The majority of respondents (84%) reported that they were at risk of getting TB. Almost half (49%) of the participants mentioned that they would feel compassionate and desire to help those infected with TB. However, another half of the respondents also had some stigmatizing attitude towards TB patients (i.e. they fear them, no particular feeling, or stay away from them).

**Table 5:** Attitudes of the respondents to TB.

Variable	Responses	Frequency	Percentages
How serious is TB?			
	Serious	411	98.1
	Not very serious	8	1.9
Is TB a public health problem in Bhutan?			
	Yes	382	91.4
	No	36	8.6
Do you think you can get TB?			
	Yes	352	83.8
	No	68	16.2
What would be your reaction on getting TB?			
	Fear	227	54.2
	Surprise	66	15.8
	Shame	2	0.5
	Embarrassment	7	1.7
	Sadness/hopelessness	61	14.6
	Others	56	13.4
What are your feelings about people with TB?			
	I feel compassion and desire to help	206	49.2
	I feel compassion but I tend to stay away from these people	92	22.0
	It is their problem and I can’t get TB	10	2.4
	I fear them because they may infect me	21	5
	I have no particular feelings	84	20.1
	Others	6	1.4
How are TB patients treated in community?			
	Most people reject him or her	47	11.2
	Most people are friendly but they generally try to avoid him or her	208	49.5
	The community supports and helps him or her	151	36.0
	Others	14	3.3

COR- crude odds ratio; AOR- adjusted odds ratio; CI- confidence interval.

Table 6 summarizes the factors associated with a good attitude. Overall, 93% of the respondents had a good attitude towards TB patients. Compared to male, the female had a significantly good attitude towards TB patients. (AOR 2.4: 95% CI 1.02–5.62; p-value 0.045).

**Table 6:** Factors associated with good attitude on TB in Samtse College of Education.

		Attitude score		Univariate analysis		Multivariate analysis	
Variables		Poor, n (%)	Good, n (%)	COR (95% CI)	p-value	AOR (95% CI)	p-value
**Attitude (N = 420)**		29 (6.9%)	391 (93.1%)	** **	** **	** **	** **
**Age (years)**							
	<23 years	14 (48.3)	222 (56.8)	1.4 (0.66–3.0)	0.38	1.22 (0.49–3.0)	0.67
	≥ 23 years	15 (51.7)	169 (43.2)	Ref		Ref	
**Sex**							
	Female	9 (31)	214 (54.7)	2.69 (1.19–6.05)	0.02	2.4 (1.02–5.62)	0.045*
	Male	20 (69)	177 (45.3)	Ref		Ref	
**Education**							
	Junior college	19 (65.5)	257 (65.7)	1.01 (0.46–2.23)	0.98	0.85 (0.3–2.43)	0.77
	Senior college	10 (34.5)	134 (34.3)	Ref		Ref	
**Marital status**							
	Single	25 (86.2)	355 (90.8)	1.58 (0.52–4.79)	0.42	Ref	
	Married/divorced	4 (13.8)	36 (9.2)	Ref		0.83 (0.21–3.26)	0.794
**Previously treated for TB**							
** **	Yes	3 (10.3)	26 (6.7)	Ref		Ref	
** **	No	26 (89.7)	365 (93.3)	1.62 (0.46–5.71)	0.45	1.61 (0.44–5.96)	0.475
**Family treated for TB**							
** **	Yes	3 (10.3)	80 (20.5)	Ref		Yes	
** **	No	26 (89.7)	311 (79.5)	0.45 (0.13–1.52)	0.2	0.44 (0.13–1.54)	0.199

*p-value <0.05

COR- crude odds ratio; AOR- adjusted odds ratio; CI- confidence interval; Junior years -First year, second year and third year; Senior Years-Final year, Post graduate, Master of Education

### Practices of trainees towards TB

The practice of patients on TB is shown in Table 7. The mean score for the practice was 1.33±0.59 (Range:0–2). A large portion of the respondents (88.1%) said that they would first visit the hospital if they had symptoms suggestive of TB. Interestingly 6% responded that they would visit medical shops or traditional healers. Eighty percent of the study participants reported that they would consult a doctor to discuss TB symptoms. However, 26% reported that they would talk about TB symptoms to close friends and 12% mentioned that they would talk to the spouse. The overall good practices related to TB was 39% and was not associated with factors in multivariable analysis (Table 8).

**Table 7:** Practice of respondents (n = 420).

Responses		Frequency	Percentages
Where would you first go if you had TB symptoms?			
	Hospital	370	88.10
	Medical shops	28	6.67
	Traditional healers	13	3.10
	Others	9	2.14
Whom will you talk about TB symptoms to?			
	Doctor	337	80.2
	Spouse	52	12.4
	Parents	193	46.0
	Children	34	8.1
	Other family members	80	19.1
	Close friend	111	26.4
	No one	8	1.9
	Others	15	3.6

**Table 8:** Factors associated with good practise on TB in Samtse College.

Practice score				Univariate analysis		Multivariate analysis	
Variables		Poor, n (%)	Good, n (%)	COR (95% CI)	p-value	AOR (95% CI)	p-value
**All respondents (N = 420)**		255 (60.7)	165 (39.3)				** **
**Age (years)**							
	<23 years	149 (58.4)	87 (52.7)			Ref	
	≥ 23 years	106 (41.6)	78 (47.3)	1.26 (0.85–1.87)	0.25	1.22 (0.76–1.96)	0.41
**Sex**							
	Female	136 (53.3)	87 (52.7)	Ref		Ref	
	Male	119 (46.7)	78 (47.3)	1.02 (0.69–1.52)	0.9	1 (0.66–1.53)	0.98
**Education**							
	Junior college	172 (67.5)	104 (63)	Ref		Ref	
	Senior college	83 (32.5)	61 (37)	1.22 (0.81–1.83)	0.352	1.08 (0.65–1.82)	0.76
**Marital status**							
	Single	232 (91)	148 (89.7)	Ref		Ref	
	Married/divorced	23 (9)	17 (10.3)	1.16 (.6–2.24)	0.66	0.99 (0.46–2.13)	0.99
**Previously treated for TB**							
** **	Yes	21 (8.2)	8 (4.9)	Ref		Ref	
** **	No	234 (91.8)	157 (95.1)	1.76 (0.76–4.08)	0.186	1.77 (0.75–4.17)	0.19
**Family treated for TB**							
** **	Yes	51 (20)	32 (19.4)	Ref		Ref	
** **	No	204 (80)	133 (80.6)	1.04 (0.63–1.7)	0.879	1 (0.61–1.66)	1

COR- crude odds ratio; AOR- adjusted odds ratio; CI- confidence interval; Junior years -First year, second year and third year; Senior Years-Final year, Post graduate, Master of Education

## Discussion

In the present study, we assessed the knowledge, attitude and practices on TB among the teacher trainees of the Samtse College of Education in Bhutan. Most participants heard about TB from family and friends. The study showed a low level of TB knowledge on causes, symptoms, diagnosis and treatment. Trainees in the junior years of college and those with the history of being treated for TB were significant correlates of the knowledge level. Female trainees were more likely than male trainees to have a positive attitude towards TB.

The most common source of information on TB was from friends, family and the teachers. The source of information varies among countries with textbooks and online websites in China [23], electronic media in Bangladesh [24, 25], posters/leaflets in Korea [26] to health workers and radios in Ethiopia [27, 28]. These differences in the sources of information could be due to differences in the participants, education level as well as the social background. The plausible reason for this finding in our study could be attributed to the cohesiveness of family and friends in Bhutan where it is mainly a joint family. Teachers play a significant role in schools particularly those with the boarding facilities. They are the first contact of students and play a pivotal role in the day-to-day running of the school. Therefore, educating teachers about TB can be helpful on two fronts. Firstly, they can disseminate TB related information to the students. Secondly, they will aid in the early identification of TB cases in school and prompt referral to hospitals for appropriate management. Despite a substantial increase of internet users in Bhutan in recent years [29], it was the least mentioned source of information for TB. The Ministry of Health of Bhutan should use popular social media like Facebook to disseminate information on the prevention and symptoms of TB.

Study participants recognised cough for more than two weeks as the commonest symptom of TB. This finding is comparable to a study in Ethiopia [27] but contrasts the findings from China and Nigeria [23, 30]. Generally, cough is the first and most common symptoms of TB [31]. The fact that cough being mentioned as a common symptom of TB is significant as it would make a positive impact on the health-seeking behaviour of the person. The majority of the participants knew that TB was commonly spread by coughing [89%]. Similar findings were reported in other studies as well [27, 32]. Frequent coughing is associated with the infectivity of TB [33] and recognition of this symptom could facilitate in seeking early treatment and care thereby reducing transmission in the community.

Risk factors of TB include HIV infection, malnutrition, overcrowding and diabetes mellitus [34]. However, in this study majority mentioned only smoking to be a risk factor for TB. DM and HIV were mentioned by only 3% and 6% to be the risk factor for TB. With increasing cases of DM [35] and HIV cases [36] in Bhutan, the TB burden is expected to increase unless timely screening and preventive actions for TB are taken. The majority of the participants mentioned that TB drugs should not be discontinued once patient feels well as it would lead to development of drug resistant TB. Defaulting treatment [37, 38] and non-adherence [39] are the main reasons for TB treatment failure and development of drug resistant. The findings in our study correlates with an earlier study in Bhutan which reported low default cases [40].

The trainees who were previously treated for TB had better knowledge than those who had no history of TB. This is in concurrent with the findings from other studies [41]. This could be due to the health education of TB patients by the counsellors or TB in-charges in health centres during treatment and subsequent follow-ups. Education level has been observed to be significantly associated with knowledge level, with senior students doing well compared to junior students [42]. However, our study contradicts the findings from other studies. The exact reason is not known. It could be due to lesser participation by the senior trainees in the health education programs due to their busy class schedules.

The overall knowledge score on TB cause, symptoms, diagnosis and treatment in this study was low. One of the plausible reasons could be lower engagement during the health education due to higher priorities for their studies. In addition, acute respiratory infections (ARI) are common in Bhutan which presents similar to TB. This could have led to confusion between TB and ARI. The knowledge on TB has also been found low as well in China [23], Bangladesh [24], Nigeria [30], Ethiopia [43] and India [44]. And these are some of the high TB burden countries [45]. One of the main reasons could be due to limited knowledge on TB leading to delayed health-seeking behaviour and late diagnosis.

In this study, the participants considered TB to be a public health problem in Bhutan and that they were at risk of getting the disease. Moreover, half of the participants reported that they would be overcome with fear if they were diagnosed with TB. Similar findings have been reported in another study [27]. These feelings could be due to the long duration of treatment, the cachexic nature of the disease as well as due to the risk of transmission of the disease to the family members. The study showed that female trainees have good attitudes towards TB cases compared to male trainees. This is consistent with studies from other countries [42]. In Bhutan, women are culturally responsible for taking care of the household works and care of the patients. This could be the reason for female having better attitude towards TB than male. However, a study in Ethiopia showed that female have a poor attitude towards TB cases compared to male [46]. The observed differences could be due to different social and cultural backgrounds.

Majority of respondents mentioned that they would visit hospital and talk to doctors about their symptoms. However, few participants mentioned that they would visit traditional healers as reported in another study [27]. This could be due to a strong cultural influence in Bhutan as shown in a study [9]. This calls for a need to educate traditional healers on symptoms of TB, so that timely referrals of suspected TB patients to the health centres can be done. Moreover, health education on TB among the trainees should be conducted time to time to change the health seeking behaviour as well as their knowledge on TB.

### Recommendations

Although TB is a public health problem in Bhutan, the symptoms of the disease and its transmission are not taught in schools. Combatting any form of public health problem requires the involvement of all stakeholders including the Ministry of Education and traditional healers. Including TB and other important public health infectious diseases in the school curriculum would help in improving the knowledge and health seeking behaviour of students as well as dessimination of information among the community by them. Secondly, there should be a regular refresher course on TB for teachers. Finally, traditional healers should be educated to identify the common signs and symptoms of TB for appropriate referrals to hospitals.

### Limitations of the study

There are a few limitations to this study. Firstly, a causal relationship cannot be established due to the cross-sectional study design. Secondly, the information was collected using self-administered questionnaire. The honesty and the seriousness of the respondents to the questions are difficult to access and validate. Fourthly, smoking and alcohol use were likely to be under-reported as a result of social desirability. Lastly, since this study was conducted in a higher educational institute, results cannot be generalized to the general population.

## Conclusion

Knowledge on TB cause, symptom and treatment and prevention were poor especially among trainees in senior years of college and those who had never suffered from TB. The attitude towards TB was good, especially among the female trainees. However, the overall practice was poor among the participants. We need innovative methods of sensitization and dissemination of information on TB. The teachers can be an important source of information to the students and help in the early identification of suspected cases of TB. Therefore, the Ministry of Health in collaboration with the Ministry of Education should sensitise the teachers and include a syllabus on a few public health problems of Bhutan to create awareness on it.

## Supporting information

S1 Material (DOC)

S2 Material (DTA)
