# Supplementary material for: Assessment of knowledge, attitude and practice on tuberculosis among teacher trainees of Samtse College of Education, Bhutan
Source: PLoS One. 2020 Nov 6;15(11):e0241923. doi: 10.1371/journal.pone.0241923 (PMC7647099; doi:10.1371/journal.pone.0241923)
Supplement: S1 Material — (DOC) [file pone.0241923.s001.doc]

**Structured questionnaire for assessing the knowledge, attitude and practice on tuberculosis (TB) among the teacher trainees of Samtse College of education.**

Section 1: Socio-demographic information

| **Sl .no** | | **Questions** | **Response** | **Remarks** |
| --- | --- | --- | --- | --- |
| 101 | | What is your age in years? | …………… years |  |
| 102 | | What is your sex? | 1. Female 2. Male 3. Others |  |
| 103 | | What is your marital status? | 1. Unmarried 2. Married 3. Divorced 4. Widowed 5. Others (specify)………………… |  |
| 104 | | What year are you studying? | 1. First year 2. Second year 3. Third year 4. Final year 5. PgDE 6. Masters 7. Others….. |  |
| 105 | | Do you/ did you ever smoke? | 1. Never smoked 2. Past smoker 3. Current smoker |  |
| 106 | | Do you/ did you ever drink alcohol? | 1. Never drank 2. Past user 3. Current user |  |
| 107 | | Have you been treated for TB before? | 1. Yes 2. No |  |
| 108 | | Has anyone of your family members suffered from TB? | 1. Yes 2. No |  |
| **Section 3: Knowledge on TB** | | | | |
| **Sl. no** | **Questions** | | **Response** | **Remarks** |
| 301 | Where did you first hear of TB? (tick all that applies) | | 1. Newspapers/magazines 2. Radio 3. TV 4. Brochures, posters and other printed materials 5. Health workers 6. Family, friends, neighbours and colleagues 7. Religious leaders 8. Teachers   10. Social media |  |
| 302 | What causes TB? | | 1. Virus 2. Bacteria 3. Fungus 4. protozoa 5. supernatural 6. don’t know |  |
| 303 | What are the symptoms of **lung TB** (Tick multiple answers)? | | 1. Cough for more than 2 weeks 2. Fever 3. Loss of Weight 4. Blood in sputum 5. Chest pain 6. Loss of appetite 7. Fatigue 8. Abdominal pain 9. Rash 10. Others 11. Don’t know |  |
| 304 | How is TB transmitted? | | 1. Through handshakes 2. Through the air when a person with TB coughs or sneezes 3. Through sharing dishes 4. Through eating from the same plate 5. Through touching items in public places (doorknobs, handles in transportation, etc.) 6. Do not know. 7. Others |  |
| 305 | How can you prevent TB? | | 1. Avoid shaking hands 2. Covering mouth and nose when coughing or sneezing 3. Avoid sharing dishes 4. Washing hands after touching items in public places 5. Closing windows at home 6. Through good nutrition 7. By praying 8. Do not know |  |
| 306 | What is the **most common** method of lung TB diagnosis? | | 1. Blood test 2. Urine and stool test 3. Chest X-ray 4. Sputum test 5. Don’t know |  |
| 307 | Is TB vaccine currently available? | | 1. Yes 2. No 3. Don’t know |  |
| 308 | How long does it take to treat TB? | | 1. 1- 2 weeks 2. 1-2 month 3. 6 -8 months 4. 1-2 years 5. Don’t know |  |
| 309 | Who can be infected with TB? | | 1. Anybody 2. Only poor people 3. Only homeless people 4. Only alcoholics 5. Only drug users 6. Only people living with HIV/AIDS 7. Only people who have been in prison 8. Don’t know |  |
| 310 | Can TB affect organs other than lungs? | | 1. Yes 2. No 3. Don’t know   2 = Don’t know |  |
| 311 | Should the TB patient discontinue drugs once he feels well? | | 1. Yes 2. No 3. Don’t know |  |
| 312 | What happens if a patient discontinues TB drugs? | | 1. Patient gets better 2. Patient develops drug resistant TB 3. Don’t know |  |
| 313 | What could increase the risks of getting TB (tick multiple answers)? | | 1. Smoking 2. Drinking alcohol 3. Malnutrition 4. HIV 5. Diabetes 6. Don’t know |  |
| 314 | Can a person who was previously treated for TB get infected again? | | 1. Yes 2. No 3. Don’t know |  |
| **Section 4: TB attitudes** | | | | |
| 401 | How serious is tuberculosis in your opinion? | | 1. Very serious 2. Somewhat serious 3. Not very serious |  |
| 402 | Is Tuberculosis a public health problem in Bhutan? | | 1. Very serious 2. Somewhat serious 3. Not very serious |  |
| 403 | Do you think you can get TB? | | 1. Yes 2. No   No |  |
| 404 | What would your reaction be if you get TB? | | ………   1. Fear 2. Surprise 3. Shame 4. Embarrassment 5. Sadness or hopelessness 6. Other: |  |
| 405 | Which statement is closest to you’re feeling about people with TB disease? (Tick one please) | | 1. I feel compassion and desire to help 2. I feel compassion but I tend to stay away from these people 3. It is their problem and I can’t get TB 4. I fear them because they may infect me 5. I have no particular feelings 6. Others: please explain………………………………………………………. |  |
| 406 | How is a person who has TB usually regarded or treated in your community? | | 1. Most people reject him or her 2. Most people are friendly but they generally try to avoid him or her 3. The community supports and helps him or her 4. Others: please explain………………………………………. |  |
| Section 5: Practice on TB | | | | |
| 501 | Where would you go if you had TB symptoms? | | 1. Hospital 2. Medical shops/pharmacy 3. Traditional healer 4. Others: |  |
| 502 | Who would you first talk about your illness if you had TB symptoms? (check all that are mentioned) | | 1. Doctor or other medical workers 2. Spouse 3. Parents 4. Children 5. Other family members 6. Close friend 7. No one 8. Others: |  |

Thank you for taking the time to answer the questions………….
